# A deep feature fusion network for fetal state assessment

**DOI:** 10.3389/fphys.2022.969052

**Published:** 2022-11-30

**Authors:** Yahui Xiao, Yaosheng Lu, Mujun Liu, Rongdan Zeng, Jieyun Bai

**Affiliations:** ^1^ Guangdong Provincial Key Laboratory of Traditional Chinese Medicine Informatization, Department of Electronic Engineering, College of Information Science and Technology, Jinan University, Guangzhou, China; ^2^ College of Science and Engineering Jinan University, Guangzhou, China

**Keywords:** cardiotocography, computer-aided diagnosis algorithm, feature fusion network, fetal state assessment, convolutional neural network

## Abstract

CTG (cardiotocography) has consistently been used to diagnose fetal hypoxia. It is susceptible to identifying the average fetal acid-base balance but lacks specificity in recognizing prenatal acidosis and neurological impairment. CTG plays a vital role in intrapartum fetal state assessment, which can prevent severe organ damage if fetal hypoxia is detected earlier. In this paper, we propose a novel deep feature fusion network (DFFN) for fetal state assessment. First, we extract spatial and temporal information from the fetal heart rate (FHR) signal using a multiscale CNN-BiLSTM network, increasing the features’ diversity. Second, the multiscale CNN-BiLSM network and frequently used features are integrated into the deep learning model. The proposed DFFN model combines different features to improve classification accuracy. The multiscale convolutional kernels can identify specific essential information and consider signal’s temporal information. The proposed method achieves 61.97%, 73.82%, and 66.93% of sensitivity, specificity, and quality index, respectively, on the public CTU-UHB database. The proposed method achieves the highest QI on the private database, verifying the proposed method’s effectiveness and generalization. The proposed DFFN combines the advantages of feature engineering and deep learning models and achieves competitive accuracy in fetal state assessment compared with related works.

## 1 Introduction

Many studies confirm that fetal hypoxia and acidosis are more likely to occur during childbirth, leading to fetal asphyxia, brain damage, and even death (Muccini et al., 2022), (Kanagal and Praveen, 2022), (Giussani, 2021). Continuous fetal monitoring during birth is crucial for detecting early signs of fetal hypoxia and preventing irreversible damage. CTG (cardiotocography) is a combined recording of fetal heart rate (FHR) and uterine contractions (UC). These time-series signals comprise the features of fetal state. When FHR features indicative of fetal oxygen deficiency are identified early, they can aid in fetal state prediction (Gupta et al., 2022), (Al-Yousif et al., 2021) and decrease respiratory acidosis in newborns and fetal brain injury (Castro et al., 2021), (Miller et al., 2021), (Gunaratne et al., 2022). CTG is sensitive in predicting the acid-base balance of fetuses but lacks specificity in identifying fetal acidosis and neurological disorders. Due to the complexity of CTG signals, visual interpretation based on guidelines result in diagnostic errors. Additionally, owing to observer variability, the false-positive rate of CTG is relatively high, leading to an increase in unnecessary Cesarean deliveries (Garabedian et al., 2017), (Ogasawara et al., 2021). The computerized data-driven analysis of CTG can assist obstetricians in reducing subjective errors and making objective medical decisions. There are two classification methods for CTG signals: machine learning and deep learning (Georgieva et al., 2019).

Machine learning identifies essential morphological features by imitating obstetricians’ inspection techniques (Nunes and Ayres-de Campos, 2016). Baseline, acceleration, deceleration, and variability are visual morphological features that represent the macroscopic aspects of FHR pattern (Akkanapalli and Mudigonda, 2022). Furthermore, several statistical approaches are used with machine learning methods to recognize potential features of CTG signal (Ponsiglione et al., 2021). On the one hand, there are several signal-based approaches as follows. Nonlinear features, such as Approximation Entropy (ApEn) (Pincus, 1995), Sample Entropy (SampEn) (Richman et al., 2004), and Lempel Ziv Complexity (LZC) (Lempel and Ziv, 1976), have been employed as diagnostic features primarily for analyzing the nonlinearity and complexity of FHR signal. Fetal heart rate variability (FHRV) offers essential information on acidosis during delivery (Gatellier et al., 2021). Long-Term Variability (LTV) and Short-Term Variability (STV) have been developed mainly for FHRV analysis (Malik, 1996). On the other hand, transform-based methods such as empirical mode decomposition, discrete wavelet transform, and Fourier transform have been applied to extract implicit CTG features (Cömert et al., 2018b). Fetal state assessment also utilizes the features derived from fast Fourier transform and continuous wavelet transform (Bursa and Lhotská, 2017).

Machine-learning algorithms are applied to classify fetal states after features are extracted and selected. Several classifiers have been used, such as support vector machine (SVM), logistic regression, K-nearest neighbors, random forest, and decision tree. Karabulut and Ibrikci. (2014) classified CTG recordings using a decision tree. Spilka et al. (2016) categorized fetal states by adopting a sparse subset of features. Likewise, Subasi et al. (2020) conducted a study with the same purpose while using more machine learning methods. Differently, Cömert and Kocamaz. (2016b) sought to categorize hypoxic fetuses. Cömert et al. (2018b) assessed fetal state through SVM. They proposed an innovative image-based time-frequency feature extraction method (IBTF) (Cömert et al., 2018a). Zeng et al. (2021) used time-frequency features and an ensemble cost-sensitive SVM classifier to classify CTG recordings. Nevertheless, machine learning algorithms involve intricate feature engineering. The model’s performance is primarily determined by the quality of feature engineering, which has a heavy workload and is prone to ignoring correlations between features.

Deep learning is a form of sophisticated machine learning that employs neural networks. Deep learning does not require feature extraction and selection, whose models extract useful features automatically by training data. Li et al. (2018) and Ogasawara et al. (2021) compared and analyzed the performance of convolutional neural network (CNN) and traditional machine learning algorithms for fetal state assessment. Their studies indicated that CNN algorithms outperformed conventional machine learning algorithms. Petrozziello et al. (2018) compared the performance of RNN and CNN in assessing fetal states, and their research suggested that CNN was more advantageous. Cömert and Kocamaz. (2018) proposed using a short-time Fourier transform to convert a signal into a visual for fetal state evaluation through CNN. Zhao et al. (2019b) combined recursive graph and CNN in order to turn signals into images that could be used to categorize fetal states. It was shown that transforming signals into images and processing them was a more effective way of predicting fetal hypoxia than merely processing the signals. Das et al. (2018) then presented a Long Short-Term Memory (LSTM) network to adjust the weights of normal and pathological recordings and improve detection accuracy. Ogasawara et al. (2021) employed CNN and LSTM architecture for analyzing CTG time series. Liu et al. (2021) proposed a CNN-BiLSTM network based on attention to obtaining the complex nonlinear spatial and temporal relationships of FHR. However, using a single-scale convolution kernel in CNN may neglect some of the signal’s latent and timing information. Unlike traditional CNN, the Multiscale Convolutional Neural Network (MSCNN) network retains global and local information synchronously. Moreover, MSCNN is capable of increasing the accuracy of medical image segmentation and provides an effective solution (Teng et al., 2019). Most studies use single feature engineering or deep learning. Clinicians are more likely to base their diagnosis on physiological parameters, given the complexity of physiological phenomena influencing fetal heart rhythm. Computer-aided CTG analysis can be a potential solution for improving CTG interpretation accuracy (Sbrollini et al., 2017).

Toward accurate and practical fetal state assessment, a feature fusion network is introduced to capture the complex features frow CTG signals. The chief contributions are summarized as follows. 1) As far as we know, this work is the first to use a deep feature fusion network (DFFN) that combines a multiscale CNN-BiLSTM model with linear and nonlinear features to improve the classification performance. 2) The multiscale CNN-BiLSTM model simultaneously derives spatial features and temporal information from CTG signals to capture complex fetal vital signs. 3) We construct the JNU-CTG database and use it to validate the generalizability of the proposed method. Compared to other researches, the present method has the best generalization performance.

## 2 Materials and methods

The public CTG database CTU-UHB and the private CTG database Jinan University cardiotocography (JNU-CTG) are employed to demonstrate the validity of methods. We propose a novel DFFN for fetal status assessment. A multiscale CNN-BiLSTM network extracts spatial and temporal information from FHR signal. The multiscale CNN-BiLSM features combined with linear and nonlinear features is used to classify fetal states.

### 2.1 Database description

In this study, we use 552 recordings from the public database and 784 recordings from the private database for fetal state assessment. There are two types of recordings: normal and pathological. The recordings with pH < 7.15 are considered pathological, while the rest are considered normal. CTU-UHB is unable to provide UC signals of sufficient quality for this experiment. This problem is also mentioned in the study of Zeng et al. (Zeng et al., 2021), which select 469 UC signals from 552 UC signals that meet the signal quality requirements (i.e., some UC signals are available) and directly delete the missing parts of 469 UC signals, resulting in a discontinuity in the signal. For the following reasons, UC signals are not used in this study: 1) A low-quality UC signal will severely reduce classification accuracy. 2) Most current studies use FHR signals for fetal state assessment. In order to demonstrate the validity of the proposed method under the same benchmark (i.e., without UC signal), we only use FHR signal for fetal state assessment.

#### 2.1.1 The public CTG database CTU-UHB

Based on clinical and technical criteria, the 552 recordings are chosen from 9164 intrapartum recordings obtained at the University Hospital in Brno, the Czech Republic (Chudáček et al., 2014). The raw data recordings are publicly available in Physionet (https://physionet.org/content/ctu-uhb-ctgdb/1.0.0/). A summary of patient and labor outcome measure statistics is also available in the database. Table 1 lists the statistical properties of CTU-UHB database. The signal has a sampling frequency of 4 Hz and a maximum recording time of 90 min. All the records are singleton pregnancies with a signal loss of 50% or less per 30-min time window and gestational weeks longer than 36 weeks.

**TABLE 1:** The statistical properties of CTU-UHB database.

Term	Mean (Median)	Minimum	Maximum
Mother’s age (years)	29.6	18	46
Parity	0.43	0	7
Gravidity	1.43	1	11
Gestational age (weeks)	40	37	43
Gestational diabetes (True/False)	515/37		
Delivery	VB: 506	CS: 46	
pH	7.23	6.85	7.47
BE	−6.38	−26.80	−0.2
BDecf (mmol/L)	4.60	−3.40	
Apgar 1 min	8.26	1	10
Apgar 5 min	9.06	4	10
Neonate’s weight (g)	3408	1970	4750
Neonate’s sex (Female/Male)	259/293		
Signal Length (min)	60	55	95

Abbreviations: VB, vaginal birth; CS, cesarean section; BE, base excess; BDecf, base deficit in extracellular fluid.

#### 2.1.2 The private CTG database JNU-CTG

**FIGURE 1:**
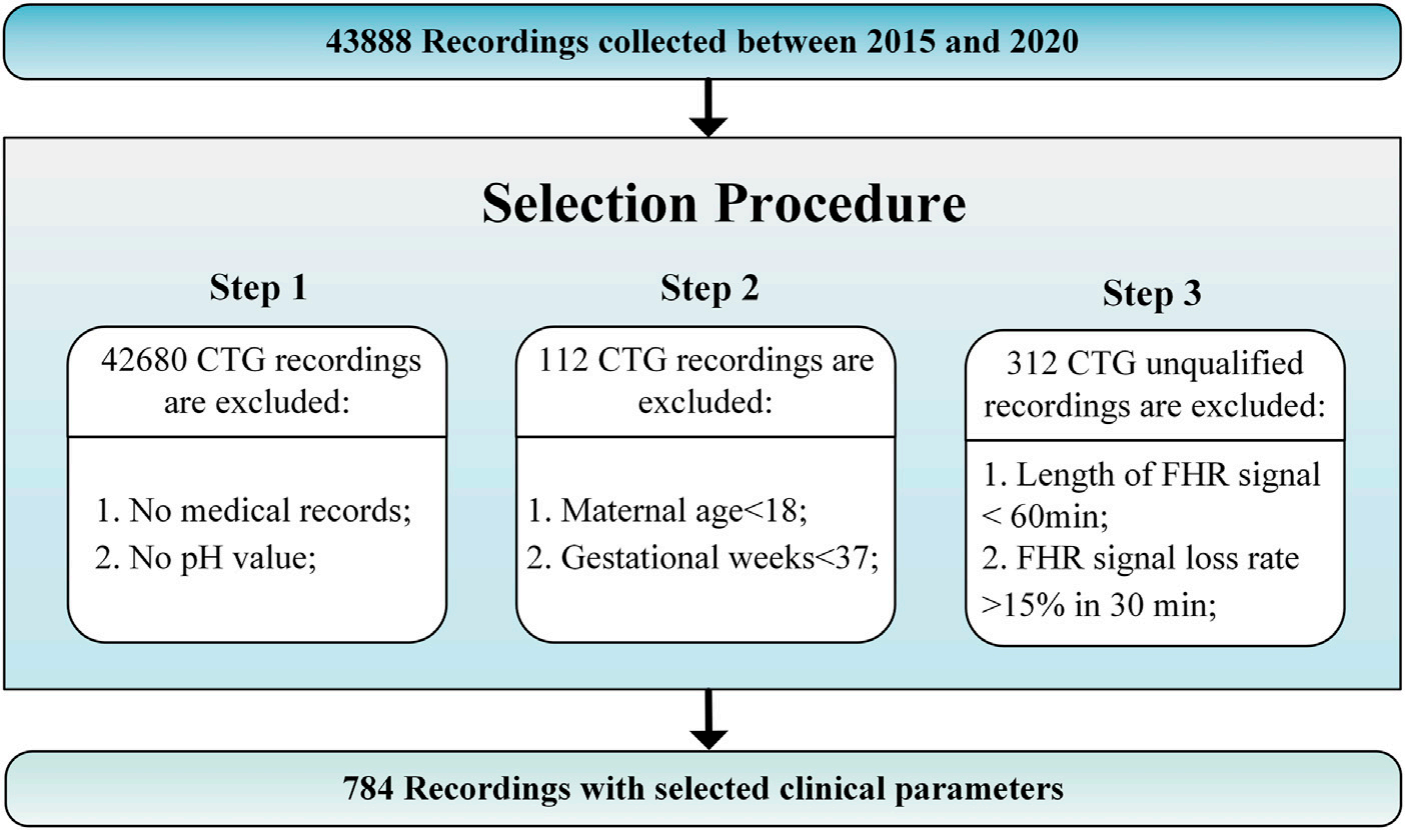
JNU-CTG database selection procedure.

The JNU-CTG database is developed to help with CTG classification and fetal state evaluation. We use JNU-CTG database to develop, test, and compare algorithms for automatic CTG analysis. Table 2 summarizes the statistical properties of JNU-CTG database. The recordings in JNU-CTG database were collected between 2015 and 2020 at the obstetrics ward of the first affiliated hospital of Jinan University. Intrapartum CTG recordings and medical records are two main components of the data. The OB TraceVue®system (Philips) stores all CTG recordings in an electronic format in a proprietary form. Furthermore, the system uses the anonymized unique identifier generated by the hospital information system to match the CTG recordings and medical records. To ensure the integrity and correctness of the database, data that does not fit clinical criteria are removed. The selection procedure is depicted in Figure 1.

**TABLE 2:** The statistical properties of JNU-CTG database.

Term	Mean (Median)	Minimum	Maximum
Mother’s age (years)	29.3	18	44
Parity	0.26	0	2
Gravidity	1.61	1	8
Gestational age (weeks)	39	37	41
Gestational diabetes (True/False)	189/595		
Delivery	VB: 549	CS: 295	
pH	7.20	6.82	7.42
Apgar 1 min	8.79	4	10
Apgar 5 min	9.87	5	10
Neonate’s weight (g)	3192	2000	4450
Neonate’s sex (Female/Male)	489/295		
Signal Length (min)	186.7	60	545.6

Abbreviations: VB, vaginal birth; CS, cesarean section.

Step 1: Unqualified recordings should be excluded according to the following guidelines. 1) Recordings that lack maternal or fetal medical records are eliminated. 2) A fetal state classification involves pH value, which determines whether CTG recording is normal or pathological. The fetal medical records without the fetal umbilical artery blood pH are excluded.

Step 2: We use the following criteria to determine which CTG recordings should be included in the final database. 1) Maternal age: Although maternal age plays a significant role in the risk of congenital disorders, there are no significant differences at delivery. The records with a low maternal age (under 18 years) are excluded since there may have been an adverse effect. 2) Gestational weeks: Fetal maturity significantly impacts the morphology and behavior of FHR before and during delivery. Thus, full-term fetuses are chosen based on their last menstrual count (37 weeks of gestation), determined by ultrasound measurements during prenatal examinations.

Step 3: CTG recordings should comply with the following rules to ensure quality. 1) The recording time for CTG is more than 60 min 2) The loss rate of fetal heart rate signals is less than 15% per 30 min.

### 2.2 Signal preprocessing

In this paper, we use the FHR signal 20 min before delivery, detect and interpolate the outliers, and finally obtain the FHR signal required for the experiment. The 20-min FHR signal is usually used to assess the state of a fetus in clinical practice since FHR signals closer to delivery are highly associated with fetal hypoxia (Chudáček et al., 2011). In our study, we use 20-min CTG recordings at the end of the first stage of labor. The signal is divided into 20-min segments, has 4,800 samples, and is sampled at a rate of 4 Hz.

**FIGURE 2:**
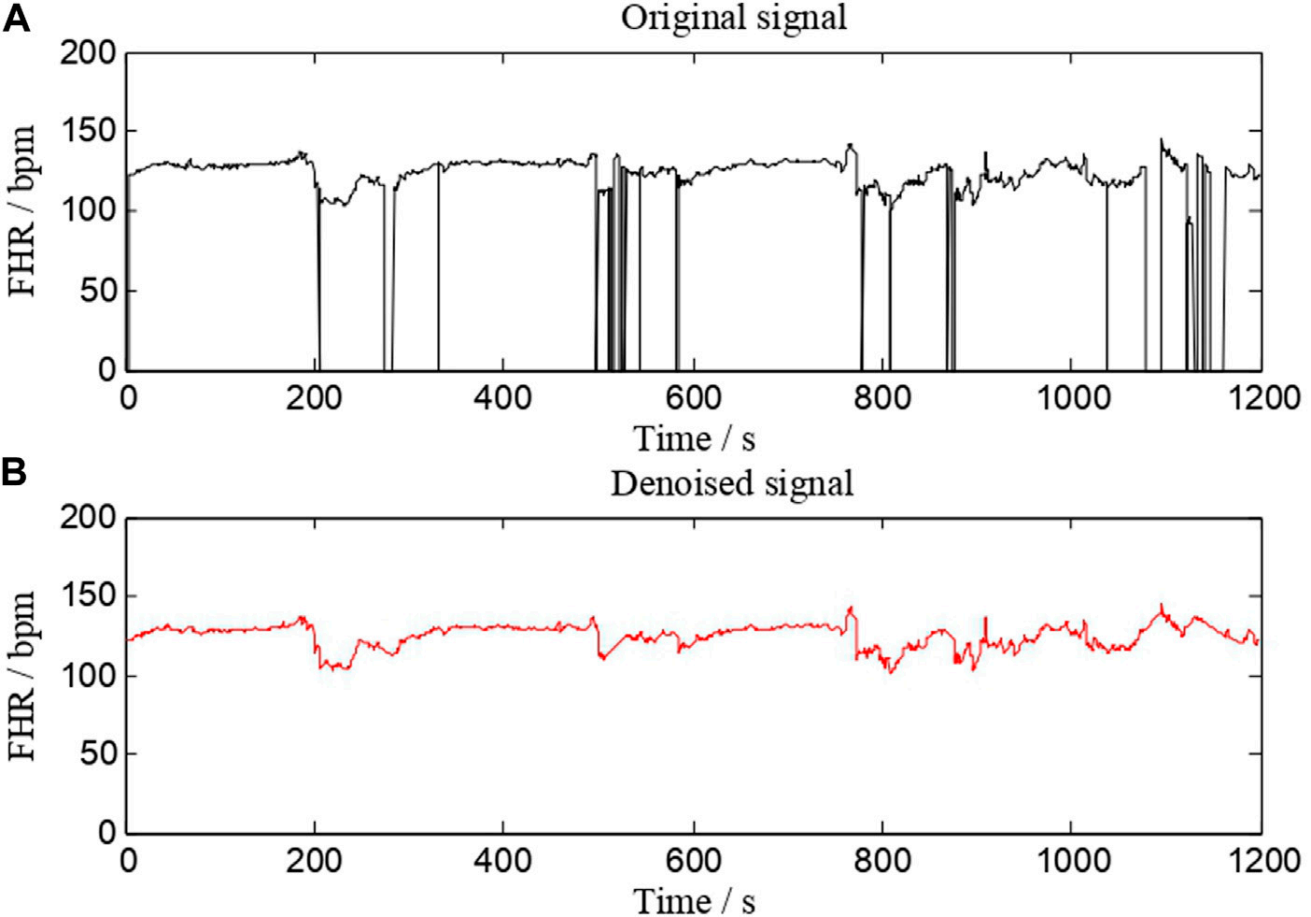
A comparison of a signal (No. 1008 FHR signal) before and after preprocessing. **(A)** is the original signal, whereas **(B)** is the denoised signal.

Preprocessing is an essential step in almost all biomedical signal processing applications. The value of extracted features and classification performance are both affected by this process. The main preprocessing processes are signal fragment selection, outlier detection, and interpolation. Our work uses the same FHR signal preprocessing method as AH del’Aulnoit et al. (de l’Aulnoit et al., 2019) for outlier detection and interpolation. These anomalous data points are recognized first, eliminated, and replaced with a linear interpolation between valid data points. Invalid data points are defined as follows. 1) The signal values are outside the acceptable range (50–220 bpm). 2) Abrupt and large deviations in FHR signal (absolute value of two adjacent points exceeding 25 bpm). A comparison of a signal (No. 1008 FHR signal) before and after preprocessing is shown in Figure 2. It suggests that this interpolation technique is capable of effectively removing noise.

### 2.3 Deep feature fusion network

**FIGURE 3:**
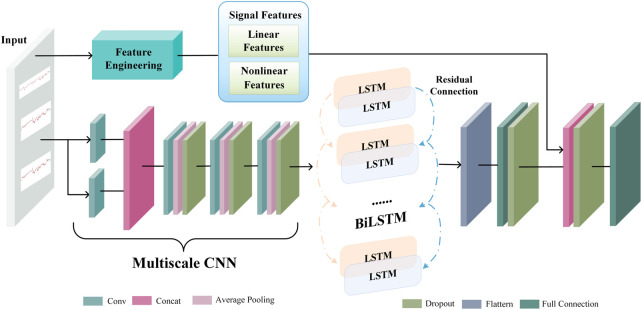
The proposed deep feature fusion network’s structure.

A deep neural network works like a feature learning process, where the initial input is abstracted step-by-step through a hidden layer. As a result, it can extract more valuable features from the original input data. An end-to-end deep learning model extracts latent representation vectors from the input FHR signal and automatically assesses the fetal status based on this information. The proposed DFFN’s structure is shown in Figure 3. The feature fusion network receives the preprocessed FHR signal as input. The complex invisible features in the FHR signal are extracted using a multiscale CNN-BiLSTM network. The multiscale CNN-BiLSTM network is used to obtain the deep neural network feature vector. The multiscale features then are spliced with the linear and nonlinear features. The fused features are transmitted to the fully connected layer. A 32-dimensional vector is extracted from the multiscale CNN-BiLSTM network *via* a fully connected layer with 32 nodes. Training and testing are relatively straightforward with the DFFN since multiscale features and feature fusion are integrated into a network. The DFFN framework consists of two stages of training. In the first stage, we obtain the optimal model for each scale, and then we extract the features of the residual block of each scale. In the second stage, the multiscale, linear, and nonlinear features are combined to train a new model. The fused features are input into a new model that learns more discriminative features for final classification. The hierarchy information in parallel is used to calculate the corresponding weight through learning. Consequently, the fused features tend to favor the features that are useful for classification, which is the weight that indicates the importance of multiscale features.

### 2.4 Extracting multiscale CNN-BiLSTM features

**FIGURE 4:**
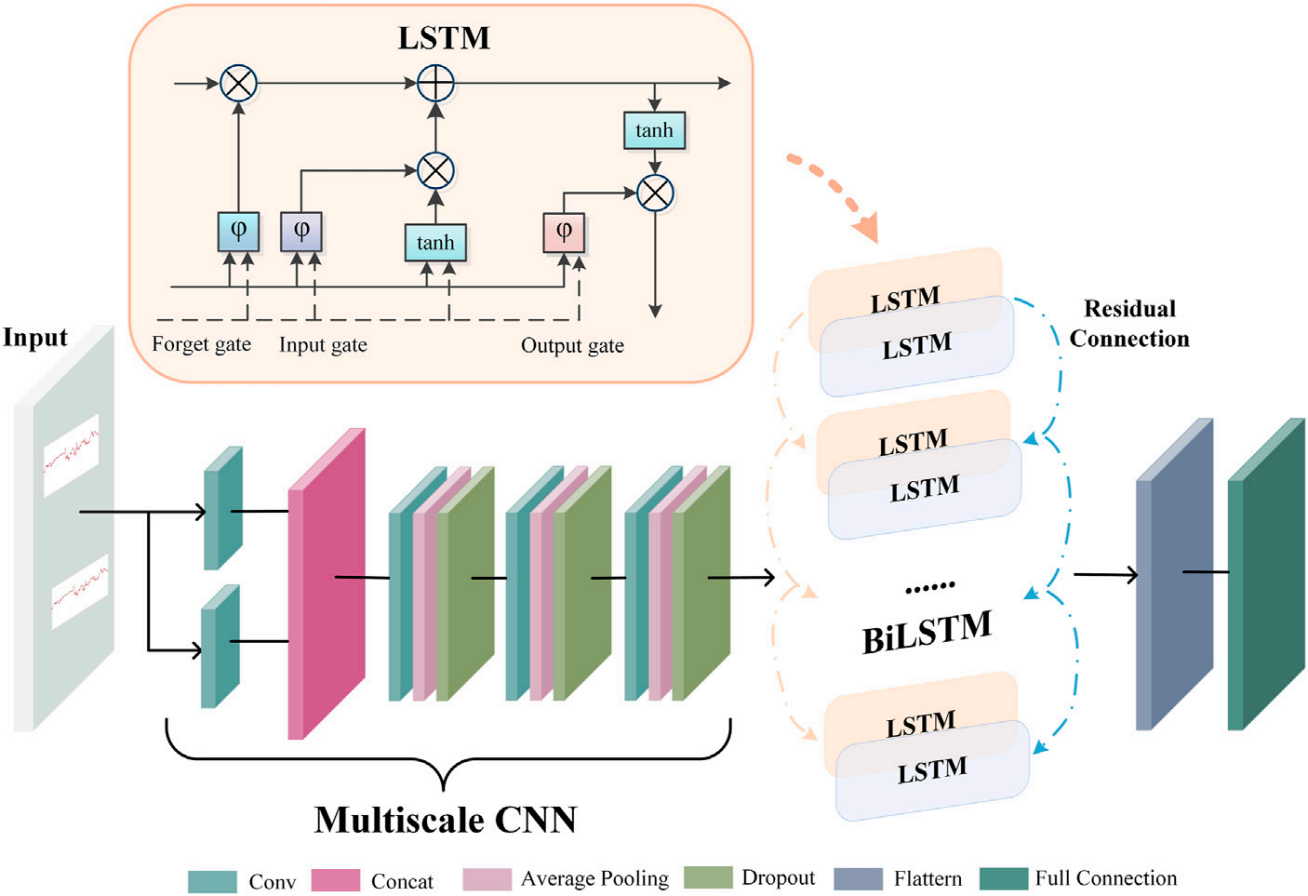
The architecture of multiscale CNN-BiLSTM network.

Figure 4 depicts the architecture of the multiscale CNN-BiLSTM hybrid network. Multiscale CNN provides a greater diversity of features than CNN. The multiscale CNN-BiLSTM network contains one multiscale layer and three convolutional layers. A batch normalization (BN), an exponential linear unit (ELU), an average pooling layer, and a dropout layer follow each convolutional layer. Dropout is valuable to the hybrid network since it reduces overfitting and improves the model’s generalization capabilities. The rate of dropout is 0.25. The hybrid neural network receives the preprocessed FHR signal as input.

FHR signals have various waveforms, resulting in huge differences between them. Therefore, it is difficult to choose a suitable convolution kernel size for the convolution operation. The single-scale convolutional kernel size limits network feature extraction. FHR signals with more global information distribution prefer larger convolution kernels, and FHR signals with more local information distribution prefer smaller convolution kernels. In multiscale layers, convolution kernels of different sizes are employed to extract different information from the FHR signal, and these operations are performed in parallel and then merged to provide a more accurate representation. In this paper, two convolution kernels of different sizes (KS = 32, 64) are used to extract features from the FHR signal, and the extracted features are dimensionally spliced to fuse features of different scales.

There is a particular type of recurrent neural network known as LSTM, which is capable of solving the vanishing gradient problem and learning long-term dependencies in neural networks. The FHR signal is a time series. The classification results will be more robust if information from past and future time points is taken into account simultaneously. In standard LSTM networks, sequences are processed chronologically, but future point-in-time information is not considered. In this paper, two independent hidden LSTM layers are combined in opposite directions as BiLSTM to compensate for this weakness. With this structure, the output layer is able to utilize information from past and future time points. The spatial features of the FHR signal are extracted using the multiscale CNN to enhance the variety of features. The temporal information features are extracted using the BiLSTM. The residual connection efficiently merges the spatial and temporal information features. The gate mechanism determines the transmission of information and can learn relevant information regarding the current information. The forget gate determines which information is irrelevant for classification and should be discarded, the input gate determines which information requires updating, and the output gate decides which information to output.

### 2.5 Linear features

It has been a consensus for a long time that linear features have been regarded as the primary indicators for evaluating FHR signals. FHR linear features are the most efficient prognostic indicators for detection of fetal distress (Cömert and Kocamaz, 2016a). The morphological and time-domain features constitute the conventionally used linear features essential for interpreting FHR signals (Cömert et al., 2018a) (Akkanapalli et al., 2022) (Fergus et al., 2018).

Morphological features are the significant indicators to ascertain fetal state in clinical practice. Obstetricians have attempted to identify specific FHR patterns that can be seen visually as morphological features (Haweel et al., 2021). Baseline, acceleration, deceleration, and variability in short and long terms represent the gross features of the FHR patterns (Cömert et al., 2018a). In this paper, they are calculated based on FIGO guidelines (Ayres-de Campos et al., 2015).

Stationary information of CTG signals is often measured with time-domain features. In clinical practice, time-domain features are easy to understand and recognize by clinicians since they have good clinical interpretability. The time-domain features are formulated as follows (Cömert and Kocamaz, 2016a) (Zhao et al., 2018). Time-domain features are physiologically closely related to physiological activities such as fetal control mechanisms, sympathetic and parasympathetic nerve activity, fetal movement, and fetal respiration (Akkanapalli et al., 2022), (Feng et al., 2018). *FHR*_mean_ denotes FHR’s mean value, whereas *FHR*_*std*_ denotes FHR’s standard deviation. *x*(*i*) is an FHR signal of length *N*, *i* = 1, … , *N*.

$$FHR_{\text{mean}}=\bar{x}=\frac{1}{N}\overset{N}{\underset{i=1}{\sum }}x\left(i\right)$$ (1)

$$FHR_{\mathrm{std}}=\sqrt{\frac{1}{N-1}\overset{N}{\underset{i=1}{\sum }}{\left(x\left(i\right)-\bar{x}\right)}^{2}}$$ (2)

LTV and STV are two kinds of FHRV. LTV is critical to determining the stability of fetal heart rate. A large LTV of the FHR signal within 10 min may contribute to the instability of the fetal intrauterine environment (Gonçalves et al., 2007). First, the FHR signal is separated into 60-s segment blocks denoted by *v*(*i*) to calculate LTV. The difference between these fragment blocks’ maximum and minimum values is then calculated as a sum. After that, *M* is used to divide this value. The *M* represents the total amount of minutes.

$$LTV=\frac{1}{M}\overset{M}{\underset{i=1}{\sum }}\left[\underset{i\in M}{\max}\left(v\left(i\right)\right)-\underset{i\in M}{\min}\left(v\left(i\right)\right)\right]]$$ (3)

The difference in FHR signal between 2.5 s connected within a minute is used to calculate STV, reflecting the FHR signal’s variability due to beat-by-beat differences (Dawes et al., 1992). Low STV has a direct correlation with the occurrence of metabolic acidemia and imminent intrauterine death (Kouskouti et al., 2018). The FHR signal is first divided into 2.5-s fragment blocks to calculate the STV. The mean *sm*(*i*) is calculated for each fragment block, consisting of 10 sample points. FHR signal frequency is 4 *Hz*. The difference between the mean *sm*(*i*) and *sm* (*i* + 1) of two consecutive fragment blocks is then calculated as the sum of the differences. Finally, *M* is divided by this value.

$$STV=\frac{1}{24M}\overset{24M}{\underset{i=1}{\sum }}|sm\left(i+1\right)-sm\left(i\right)|$$ (4)

LTI identifies a long-term irregularity. Calculate the square root of the sum of *sm*(*i*) and *sm* (*i* + 1). *M* is divided by this value.

$$LTI=\frac{1}{24M}\overset{24M}{\underset{i=1}{\sum }}\sqrt{\left(sm\left(i+1\right)+sm\left(i\right)\right)}$$ (5)

The interval index, denoted by *II*, indicates FHR variability over a short period.

$$II=\frac{FHR_{\mathrm{std}}}{std\left[sm\left(i\right)\right]}$$ (6)

The absolute value of the FHR signal *x*(*i*) from the mean value of the FHR signal. *FHR*_mean_ is averaged to get *FHR*_mean AD_.

$$FHR_{\text{mean AD}}=\frac{1}{N}\overset{N}{\underset{i=1}{\sum }}|x\left(i\right)-\bar{x}|$$ (7)

The deviation between the FHR signal value *x*(*i*) and the median of the FHR signal (*x*(*N*)) is computed, followed by the median of the absolute magnitude of the deviation *FHR*_median AD_.

$$FHR_{\text{median AD}}=median\left(\left|x\left(i\right)-median\left(x\left(N\right)\right)\right|\right)$$ (8)

### 2.6 Nonlinear features

Nonlinear analysis is conducted to identify the essence of complex phenomena, effectively addressing the complexity of the FHR time series. A nonlinear approach may reveal relevant clinical information of FHR that cannot be revealed by conventional time series analyses, such as abnormalities in heart rate (Spilka et al., 2012). The methods of ApEn, SampEn, and LZC for the analysis of nonlinear time series have been found to increase the accuracy of the fetal status assessment significantly (Zhao et al., 2019a), (Usha Sri et al., 2020), (Marques et al., 2020). These features allow for the measurement of FHR variability, which is beneficial for clinically interpreting the fetal wellbeing during the final stage of delivery (Georgoulas et al., 2006).

#### 2.6.1 Approximate entropy

The degree of data disbandment in a system is calculated by ApEn. ApEn is a nonlinear parameter that measures the unpredictability and regularity of physiological time series. It is used to assess the internal complexity of time series and anticipate the possibility of new information arriving in them. A *N*-length time series indicated by *x*_*n*_ is divided by a collection of *m*-length vectors represented by *u*_*m*_(*i*). The *u*_*m*_(*i*) and *u*_*m*_(*j*) vectors are then written as $n_{i}^{m}\left(r\right)$ in terms of Euclidean sense $d\left[u_{m}\left(i\right),u_{m}\left(j\right)\right]\leq r$ . As stated $C_{i}^{m}\left(r\right)=\frac{n_{i}^{n}}{N-m+1}$ , the number is used to compute the possibility of vectors being near. Define the function: $\Phi^{m}\left(r\right)=\frac{1}{N-m+1}\sum_{i=1}^{N-m+1}\ln C_{i}^{m}\left(r\right)$ . ApEn is defined as follows.

$$ApEn\left(m,r\right)=\underset{N\to \infty }{\lim}\left[\Phi^{m}\left(r\right)-\Phi^{m+1}\left(r\right)\right]$$ (9)

#### 2.6.2 Sample entropy

For the *S*_*N*_ time series, SampEn is calculated by the same process and metrics as ApEn. It provides a quantitative measure of the complexity of time series, similar to ApEn. The fundamental difference between the two methods is that ApEn considers self-matches, whereas SampEn does not. SampEn also has fewer biases. Due to the elimination of self-matches, SampEn requires a lower computational time and is remarkably independent of signal length. Its definition is as follows.

$$SampEn\left(m,r\right)=\ln\Phi^{m}\left(r\right)-\ln\Phi^{m+1}\left(r\right)$$ (10)

The *m* and *r* parameters are set to the same values as with ApEn in our work: *m* = 4, *r* = 0.15, and *r* = 0.2.

#### 2.6.3 Lempel ziv complexity

LZC predicts recurring patterns in time series. It is applicable in the non-stationary signal. As a result, each time series may be described with fewer data. The number of patterns in the sequence is counted, and each time a new pattern emerges, the complexity value *c*(*n*) increases by one. The upper constraint on the complexity *c*(*n*) is known from the current work, which is $\lim_{n\to \infty }c\left(n\right)=b\left(n\right)=\frac{N}{\log_{a}N}$ , where *a* represents the number of distinct patterns in the time series. To address the issue of varying complexity caused by sequence length, the LZC is defined as follows.

$$LZC=\frac{c\left(N\right)}{b\left(N\right)}$$ (11)

Our experiment use a 20-min FHR signal with a rate of 4 Hz and a total data length of 4,800. *N* is set to 4,800 for calculating LZC.

### 2.7 Performance metrics

Four umbilical artery pH cutoffs are used to categorize fetuses as acidemic or non-academic: 7.05, 7.10, 7.15, and 7.20 (Castro et al., 2021). The pH value of 7.15 is determined as the threshold value in this paper after extensive research (Sholapurkar, 2020) (Singh et al., 2021). Blood with a pH of less than 7.15 is regarded as hypoxia, whereas blood with a pH of more than 7.15 is considered normal. This work uses a sigmoid function to do binary classification for fetal status assessment since its results are in two categories (hypoxia and normal). The function’s input is the integrated expression of FHR signal features *f*_*z*_. The *p* denotes the output. The function is calculated as follows. The weight matrix is *W*_*P*_, and the bias matrix is *b*_*P*_.

$$P=sigmoid\left(W_{P}\cdot f_{z}+b_{P}\right)$$ (12)

The cross-entropy cost function is the loss function in the training process. The expected output is *y*, and $\dot{y}$ is the actual output.

$$Loss=-\left(y\log\dot{y}+\left(1-y\right)\log\left(1-\dot{y}\right)\right)$$ (13)

We use the Sensitivity (SE), Specificity (SP), and Quality Index (QI) calculated from the confusion matrix to assess the proposed method’s performance. SP is the percentage of normal samples that are correctly recognized. SE measures the discriminative power of the model on hypoxic samples. QI is defined as the geometric mean of SE and SP. An unbalanced database can harm the overall performance of any classifier. The ratio of normal to hypoxic samples is about 4:1 in this study. As a result, QI is used to assess overall classification performance. These metrics are formulated as follows:

$$\mathrm{SE}=\frac{TP}{TP+FN}$$ (14)

$$SP=\frac{TN}{TN+FP}$$ (15)

$$QI=\sqrt{SE\cdot SP}$$ (16)

Where TP, FP, FN, and TN represent true positive, false positive, false negative, and true negative.

## 3 Experimental results

The proposed DFFN is built using Python, the Keras library, and TensorFlow as a backend. The model is trained and tested on a computer with a 2.60 GHz CPU, an NVIDIA GeForceRTX2080Ti GPU, and a 128 GB memory stick. Signal preprocessing is performed in MATLAB Aulnoit et al. (2019).

### 3.1 Determination of class weight and network parameters

It is generally acknowledged that neural networks contain many factors that might influence their performance. The settings are tweaked in the following method in our experiment. The network is trained for 130 epochs with an initial learning rate of 0.01, which declined by ten at 15 and 90 counts. The network is optimized using stochastic gradient descent with momentum, with the momentum set at 0.9 in this experiment. To assess the algorithm’s accuracy, we employ a 10-fold cross-validation procedure. The complete FHR signal of the CTU-UHB database is randomly divided into 10 folds. Stratified sampling is used to combine nearly the same proportion of normal and pathological samples in each fold. The training set consists of 90% of recordings (395 normal and 101 pathological), while the remaining 10% (44 normal and 12 pathological) are utilized to test the proposed approach’s performance. The process is repeated ten times, reinitializing and testing the model with a new subset of data before averaging the final findings. The weights of normal and pathological sample categorization are changed in this experiment due to data imbalance (the number of normal and pathological samples is roughly 4:1). To verify the generalization of methods, JNU-CTG database is used as an independent test dataset.

Experiments are carried out using various classification weights, as indicated in Table 3. Furthermore, QI is used as the final metric for evaluating model performance. Higher QI values indicate better performance. This experiment shows that the QI values vary for different classification weights. The model’s QI increases as the weight of normal samples decreases. The model’s QI decreases as the weights of pathological samples increase further. The proposed DFFN focuses on recognizing hypoxia FHR recordings when the weight of pathological samples increases and the detection rate of normal samples is dramatically lower. When the classification weights ratio is 0.21 : 0.79, the QI value is the highest. The DFFN with a ratio of 0.21 : 0.79 enhances the likelihood of identifying aberrant signals while preserving its capacity to detect normal signals. It maintains sensitive detection of both normal and pathological samples. As a consequence, 0.21 : 0.79 is chosen as the classification weight.

**TABLE 3:** Performance of DFFN on CTU-UHB database with different class weights.

Class weights (N:P)	SE (%)	SP(%)	QI (%)
0.22: 0.78	57.58 ± 17.06	76.32 ± 5.55	65.54 ± 11.19
**0.21: 0.79**	**61.97** ± **16.47**	**73.82** ± **5.35**	**66.93** ± **10.20**
0.20: 0.80	61.97 ± 16.47	68.57 ± 4.47	64.49 ± 9.69
0.19: 0.81	65.61 ± 19.64	65.84 ± 5.05	64.75 ± 10.42
0.18: 0.79	67.42 ± 16.62	62.19 ± 4.95	64.17 ± 8.14
0.17: 0.83	70.91 ± 18.10	55.14 ± 3.63	61.95 ± 8.46
0.16: 0.84	71.74 ± 17.30	49.89 ± 4.33	59.33 ± 8.03

Note: N represents normal samples, and P represents pathological samples.

**FIGURE 5:**
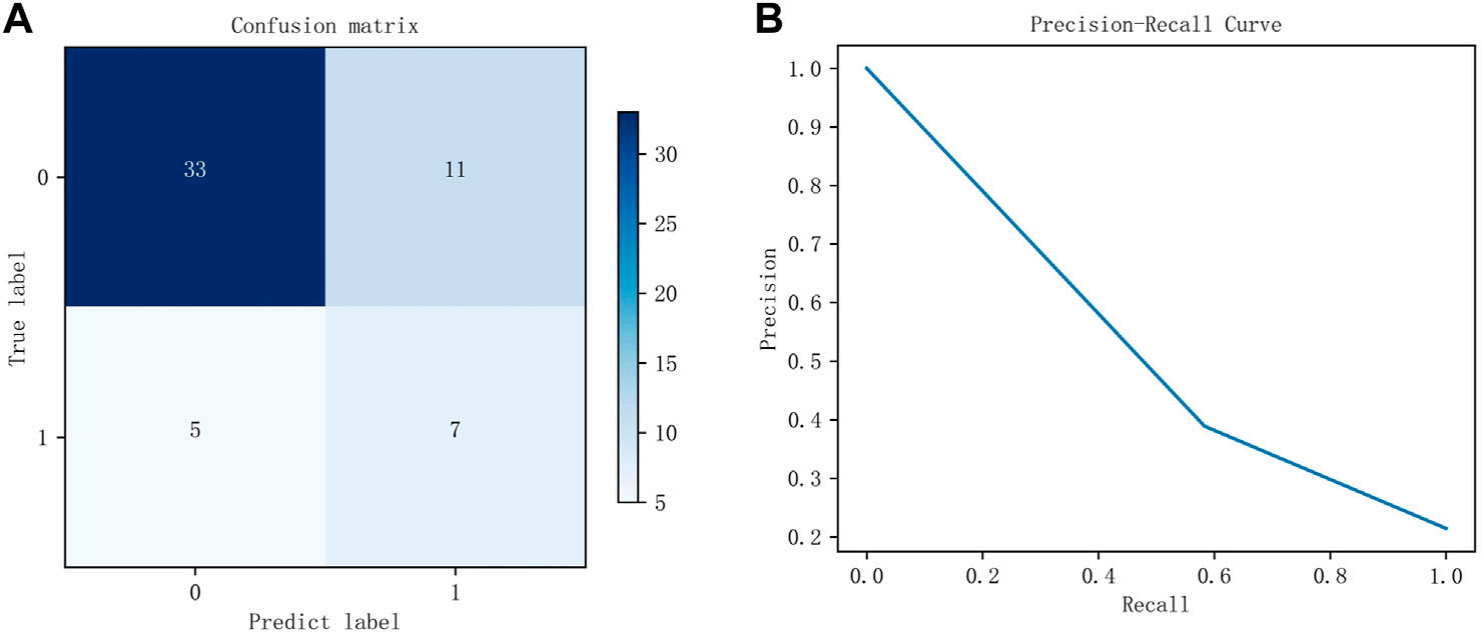
The experimental results of the test set. **(A)** is the confusion matrix using the parameters listed in Table 4, whereas **(B)** is the P-R curve using the parameters listed in Table 4.

The DFFN parameters are modified layer by layer based on the QI value. The parameters for each layer in Figure 3 are listed in Table 4. Table 4 lists the parameters for each layer in Figure 3. Figure 5 depicts experimental results obtained with the settings in Table 4. For imbalanced data sets, Precision-Recall (P-R) curves outperform receiver operation characteristic curves in comparing the performance of different models. Consequently, the P-R curve has been used to illustrate the experimental results. Figure 5A depicts the confusion matrix for the test set, whereas Figure 5B depicts the P-R curve for the test set.

**TABLE 4:** Network parameters.

Layer name	Size	Input			Output
		Number	Stride	Padding	Feature map
Signal input	−	−	−	−	4,800 × 1
Conv1	32 × 1	8	1	SAME	4,800 × 8
Conv2	64 × 1	8	1	SAME	4,800 × 8
Concat	−	−	−	−	4,800 × 16
Conv3	32 × 1	24	1	SAME	4,800 × 24
Average pooling	4 × 1	−	4	VALID	1200 × 24
Dropout	−	0.25	−	−	1200 × 24
Conv4	32 × 1	24	1	SAME	1200 × 24
Average pooling	8 × 1	−	8	VALID	150 × 24
Dropout	−	0.25	−	−	150 × 24
Conv5	32 × 1	24	1	SAME	150 × 24
Average pooling	16 × 1	−	16	VALID	9 × 24
Dropout	−	0.25	−	−	9 × 24
BiLSTM	1	9	−	−	9 × 24
Add	−	−	−	−	9 × 24
Flatten	−	−	−	−	1 × 216
Fully connection	32	−	−	−	1 × 32
Dropout	−	0.5	−	−	1 × 32
Feature input	−	−	−	−	1 × 16
Concat	−	−	−	−	1 × 48
Dropout	−	0.25	−	−	1 × 48
Fully connection	1	−	−	−	1 × 1
Sigmoid	−	−	−	−	1 × 1

### 3.2 Performance of different features

Experiments are conducted on the public CTU-UHB database to compare the outcomes of fetal state classification for different features. A SVM classifier is derived from structural risk minimization theory. It transforms the classification problem of samples into the optimization problem of classification hyperplane in the sample feature space. Table 5 compares performance utilizing SVM for linear and nonlinear features and their combinations. Linear and nonlinear features have a QI of 61.12% and 57.70%, respectively, for the evaluation index for fetal status assessment. The performance of linear features outperforms that of nonlinear features in the SVM classifier. Additionally, the QI value for their combination is 64.90%, which suggests that combining both features could increase the accuracy of fetal status assessment. And their combination achieves highest SE. The SP of linear features reaches the highest value, 80.87%, which indicates that the linear feature can discriminate hypoxic samples exceptionally well.

**TABLE 5:** Performance of SVM on CTU-UHB database.

Features	SE (%)	SP (%)	QI (%)
Linear Features	47.20 ± 15.84	80.87 ± 6.28	61.12 ± 11.38
Nonlinear Features	54.02 ± 14.08	63.28 ± 8.61	57.70 ± 7.84
Linear and Nonlinear Features	55.08 ± 16.81	78.13 ± 5.19	64.90 ± 10.66

Logistic Regression classifiers are normalized linear regression models that incorporate a logistic function based on linear regression. Table 6 shows the classification performance of different feature sets in the logistic regression classifier. The QI of linear and nonlinear features is 61.72% and 59.87%, respectively. The QI value of 63.91% indicates that combining linear and nonlinear features improves fetal state classification accuracy. The SP of linear features also reaches the highest value in logistic regression, 74.95%, indicating that linear features can distinguish hypoxic samples extremely well. In the logistic regression classifier, nonlinear features achieved the highest SE, 58.48%. This indicates the use of nonlinear features can be beneficial in identifying normal fetuses.

**TABLE 6:** Performance of Logistic Regression on CTU-UHB database.

Features	SE (%)	SP (%)	QI (%)
Linear Features	52.65 ± 19.68	74.95 ± 3.46	61.72 ± 12.28
Nonlinear Features	58.48 ± 13.61	62.38 ± 5.58	59.87 ± 7.26
Linear and Nonlinear Features	56.97 ± 17.47	73.35 ± 3.79	63.91 ± 10.20

As shown in Tables 5, 6, logistic regression classifier outperforms SVM classifier for classification using just linear or nonlinear features. SVM classification is superior to logistic regression when used with their combination.

### 3.3 Performance of various networks structures

The classification performance of different network structures on CTU-UHB database is shown in Table 7. CNN has been found to outperform traditional machine learning methods for image processing in previous studies. The CNN is capable of not only extracting low-level features and local features from the original signal, but also integrating those features into high-level features for analysis. The overall outcome of FHR signal diagnosis is closely related to some local waveforms. The purpose of CNN is to extract visible waveform features from the raw waveform signal and integrate these features into high-level features related to fetal hypoxia. Compared with CNN, multiscale CNN can increase the diversity of features. The experimental results prove that the classification performance of multiscale CNN(i.e., 65.12%) outperforms that of CNN (i.e., 63.90%). BiLSTM networks are widely used in time series forecasting and classification research because of their unique ability to capture long-term and short-term temporal relationships. The multiscale CNN-BiLSTM achieves the best performance (i.e., 65.74%) and is senstive to recognize pathlogical recrodings (i.e., 66.92%), indicating the model can integrate both spatial and temporal information features of the FHR signal to maximize the classification performance.

**TABLE 7:** Performance of different network structures on CTU-UHB database.

Network Structure	SE (%)	SP (%)	QI (%)
CNN	66.29 ± 14.46	62.65 ± 7.77	63.90 ± 8.98
Multiscale CNN	65.45 ± 12.40	65.38 ± 4.92	65.12 ± 7.94
Multiscale CNN-BiLSTM	66.29 ± 13.37	65.84 ± 5.90	65.74 ± 8.65

### 3.4 Performance of related works on two databases

We present a neural network with feature fusion to assist obstetricians in making objective clinical judgments on fetal state. In order to analyze the experimental results of this paper more comprehensively, Table 8 presents the results of a comparison between the proposed methods and previous works on the CTU-UHB database. Numerous variables, such as the FHR signal properties and the selection of signal fragments from the database, lead to varied experiment outcomes. The research evaluated in Table 8 employs the identical processing steps: signal preprocessing, feature extraction, feature selection, and final classification. To verify the validity of the proposed method, the work of (Liang and Li, 2021), (Li et al., 2018), (Zhao et al., 2019b), and (Baghel et al., 2022) are repeated in this paper. Experiments are conducted under identical settings and identical databases.

**TABLE 8:** Performance of previous works on CTU-UHB database.

Author	Method			Performance (%)		
	Extraction	Selection	Classifier	SE	SP	QI
Cömert et al. (2018b)	BFS + DWT	—	LS-SVM	57.42	70.11	63.44
Cömert et al. (2018a)	IBTF	GA	LS-SVM	63.45	65.88	64.65
Liang and Li. (2021)*	CNN	—	—	33.48	77.46	50.35
Baghel et al. (2022)*	1D-FHRNet	—	—	50.15	61.26	54.32
Zhao et al. (2019b)*	RP + CNN	—	—	54.17	61.73	57.22
Li et al. (2018)*	CNN	—	—	52.12	74.93	61.02
Ours	Multiscale CNN-BiLSTM	—	—	66.29	65.84	65.74
Ours	DFFN	—	—	61.97	73.82	66.93

Note: * is the reproduction method of this article. BFS, basic feature set; DWT, discrete wavelet transform; IBTF, image-based time-frequency features; GA, genetic algorithm; RP, recursive graph.

We employ a multiscale network to classify the fetal state and compare it to other works on the public database.

1) Comparing with (Cömert et al., 2018a), (Cömert et al., 2018b), the proposed multiscale model is more effective since it did not use complicated features. The proposed multiscale CNN-BiLSTM model has the highest SE and slightly lower SP for the same FHR signal classification criterion. The evaluation index QI is increased by 1.09% and 2.3% compared with the IBTF and BFS + DWT techniques, respectively, highlighting the hybrid model’s benefits.
2) (Liang and Li, 2021) and (Li et al., 2018), who separate the FHR signal into several sub-segments before processing the data in parallel using CNN. After that, the fetal status is determined utilizing a voting system. The difference is that (Liang and Li, 2021) utilized a system based on weighted voting. Using the same deep learning method (CNN), the QI and SE for fetal hypoxia detection of the proposed multiscale model are much superior to their method.
3) (Zhao et al., 2019b) employ recursive graphs to turn signals into images and CNN for fetal status evaluation. All the metrics of the proposed multiscale model are higher than RP + CNN, indicating that the multiscale model suggested in this study could capture the FHR signal’s hidden features more sensitively.
4) The direct input of the FHR signal is used to assess the fetal state by a neural network and automatically learn essential features in the work of (Baghel et al., 2022). We apply the same procedure and employ a multiscale model that can account for spatial features and temporal data extraction. The SP, SE, and QI of the proposed multiscale model are higher than their method, showing that our work is more accurate in fetal status classification.

We propose the DFFN, including linear and nonlinear features with the multiscale CNN-BiLSTM network. The experimental results of DFFN and other work on the public database are shown in Table 8.

1) (Cömert et al., 2018a), (Cömert et al., 2018b), utilize some time-domain, and nonlinear features. These features perform better for fetal hypoxia identification (i.e.,SE) but are less efficient for normal fetal detection (i.e.,SP). We integrate more complex features automatically retrieved by deep learning to increase the model’s capacity to recognize normal fetuses while retaining superior performance for fetal hypoxia identification.
2) In comparison to (Liang and Li, 2021), (Li et al., 2018), (Zhao et al., 2019b), and (Baghel et al., 2022), who all utilize the deep learning approach. Deep learning is sensitive for normal fetal detection but less sensitive for fetal hypoxia detection. The proposed DFFN contains both linear and nonlinear features. Therefore, the expressive capacity of DFFN and the model’s ability to identify fetal hypoxia have been improved. Meanwhile, the performance of normal fetal detection has been preserved.
3) The proposed DFFN in this study has the highest classification accuracy compared to previous fetal state assessment methods. It overcomes the constraints of a single model and compensates for the shortcomings of feature engineering and deep learning model. In addition, the performance of the proposed feature fusion approach is superior to that of the proposed multiscale CNN-BiLSTM network. The QI of the proposed DFFN method is 66.96%, 1.22% higher than the multiscale CNN-BiLSTM network.

The generalization of the proposed DFFN and multiscale CNN-BiLSTM network is tested by an independent test set of JNU-CTG database. The experimental results are shown in Table 9. The experiment is more challenging in the independent test set. However, the proposed DFFN still performs best on the test set with a QI of 53.60%. The generalization ability of the fusion network is enhanced compared to other methods. The SE and SP of the proposed DFFN method are 43.94% and 65.53%, respectively. The proposed models are capable of identifying both normal and hypoxic fetal states.

**TABLE 9:** Performance of different methods on JNU-CTG database.

Author	Method	SE (%)	SP (%)	QI (%)
Baghel et al. (2022)*	1D-FHRNet	11.97 ± 5.05	81.56 ± 3.85	30.47 ± 5.60
Liang and Li. (2021)*	CNN	27.62 ± 7.69	79.96 ± 5.00	46.29 ± 5.34
Li et al. (2018)*	CNN	27.77 ± 10.01	81.35 ± 5.63	46.44 ± 6.87
Zhao et al. (2019b)*	RP + CNN	40.05 ± 4.86	65.36 ± 4.37	50.95 ± 1.80
Ours	Linear and Nonlinear Features	21.24 ± 2.39	78.61 ± 1.24	40.78 ± 2.13
Ours	(SVM)	34.72 ± 2.16	73.59 ± 0.96	50.52 ± 1.55
Ours	Multiscale CNN-BiLSTM	37.15 ± 3.44	76.35 ± 1.49	53.18 ± 2.21
Ours	DFFN	43.94 ± 2.39	65.53 ± 2.60	53.60 ± 0.60

Note: * is the reproduction method of this article. RP, recursive graph.

## 4 Discussion

Previous studies have pointed out that imbalanced dataset is a problem for machine learning since they are biased toward majority classes and tend to miss minority class cases (Ahsan and Siddique, 2022). Therefore, we focus more on SE (i.e., the minority cases) when evaluating classification performance. We propose a DFFN model to classify CTG recordings. The model includes multiscale feature extraction, fusion, and classification and automatically fuses different features through end-to-end learning.

In this work, we integrate linear and nonlinear features. The combination of linear and nonlinear features can achieve better classification performance compared to a single feature set, as shown in Tables 5, 6. Tables 5, 6 show the performance of logistic regression and SVM on the public database. There is a relatively high accuracy rate for classifying normal fetuses but poor accuracy for classifying acidosis fetuses for two classifiers. This difference is more pronounced when experiments are conducted using private databases (see Table 9). According to Tables 5, 6, 9, SVM outperforms logistic regression with combined features on the public dataset, while on the private dataset, logistic regression outperforms SVM. It suggests that machine learning and traditional features are not very feasible. One of the limitations of machine learning is its instability. Classifiers that perform well on old data rarely perform consistently on new data, necessitating continual model development and tuning. The experimental results on the public database are presented in Tables 5, 6, 8. They demonstrate that (Cömert et al., 2018a) uses IBTF features, which can distinguish normal and acidic fetuses more accurately than other machine learning methods (combination of linear and nonlinear features, BFS + DWT). It is temporarily unable to test (Cömert et al., 2018b) and (Cömert et al., 2018a) on the private database since the essential details of their works are unavailable.

The experiments on two databases clearly demonstrate that our proposed model is superior to other deep learning-based fetal state classification models, as shown in Tables 8, 9. In the experiment of the public dataset (Liang and Li, 2021), and multiscale CNN-BiLSTM perform best at identifying normal fetuses and acidic fetuses, respectively. And DFFN has the highest QI value. The model of (Baghel et al., 2022) outperforms other methods on the private database when identifying normal fetuses, while DFFN outperforms other methods when identifying acid fetuses and has the highest QI value. Based on the experimental results of two databases, (Cömert et al., 2018a), (Zhao et al., 2019b), and DFFN are more capable of distinguishing normal and acid fetuses. Despite having good accuracy in identifying normal fetuses, the studies of (Baghel et al., 2022), (Liang and Li, 2021) and (Li et al., 2018) are grossly insufficient in identifying acid fetuses. The proposed multiscale CNN-BiLSTM network and DFFN achieve higher classification accuracy when compare to the single-scale networks used by (Zhao et al., 2019b), (Baghel et al., 2022), (Liang and Li, 2021), and (Li et al., 2018). It is attributed to the fact that many regional features in FHR signal are preserved during multiscale feature fusion process. These features are weighted and calculated as the final features of fetal status classification. (Cömert et al., 2018a), (Zhao et al., 2019b), DFFN, and multiscale CNN-BiLSTM network are better able to capture the timing-related information of FHR signals. The signal is transformed into a picture by (Cömert et al., 2018a) and (Zhao et al., 2019b), from which time-frequency features can be extracted that more accurately reflect the non-stationarity of FHR. The proposed multiscale CNN-BiLSTM network and DFFN have a BiLSTM module that extracts forward and backward information simultaneously from the FHR signal sequence. Rather than treating the data having time steps, CNN treats it as a sequence that can be read using convolutional operations. Consequently, it is difficult for CNN to acquire the time-domain features of FHR signals automatically. By incorporating BiLSTM, FHR signals can be classified more accurately and time-series features can be captured. The QI value of DFFN is higher than that of multiscale CNN-BiLSTM network on two databases. The DFFN can more precisely express the original features of signal because feature fusion realizes the complementary advantages between features.

Computerized CTG analysis can reduce the inter- and intra-observer variability caused by pattern recognition based solely on existing guidelines. However, most proposed models focus only on improving classification accuracy, ignoring the clinical relevance of parameters and the obstetrician’s decision-making mechanism. In clinical decision-making, obstetricians are more inclined to make diagnoses based on objective parameters of specific physiological significance. Obstetricians are unlikely to trust black-box deep learning model. In this study, traditional and multiscale network features are combined for the first time, maximizing fusion features and improving fetal state accuracy significantly. Morphological features, which are used in clinicians’ diagnoses, are combined in order to provide interpretability of proposed fetal status assessment model. Meanwhile, the experimental results validate the generalization of DFFN, making it more applicable in clinical practice.

We intend to integrate clinical parameters into deep learning algorithms in the future, such as maternal tachycardia and maternal pyrexia, which are collected from maternal records. Further research can include UC and FHR signals as inputs to the neural network. The more comprehensive input information may allow network models to extract more valuable features Furthermore, we hope to study our model on a larger dataset to develop a lightweight algorithm that can be applied to large-scale data. Since the two databases have similar selection criteria, further work might increase the model’s generalization using data of diverse quality.

## 5 Conclusion

This paper proposes a novel deep feature fusion network for diagnosing fetal acidosis from FHR signals. A multiscale CNN-BiLSTM hybrid network is developed to extract the signal’s temporal and spatial features adequately. In order to account for clinical physiological parameters and assessment accuracy, a feature fusion network is used to splice the multiscale CNN-BiLSM features, as well as the currently popular linear and nonlinear features. Encouraging results are obtained, with a SE of 61.97%, SP of 73.82%, and QI of 66.93% on the public database. The proposed DFFN has the highest QI value on two databases, which indicates that the proposed feature fusion model has good generalization. The experimental results on two databases show that DFFN achieves better performance than previous works. The accuracy of fetal state classification as well as the generalization of DFFN are improved by merging the FHR features from multiscale layers with the extra features. In the future, we will work to optimize the interpretability of our model as well as its accuracy and generalizability. Through these advancements, we will be able to gain a deeper understanding of particular disease state of the fetus.

## Data Availability

The CTU-UHB database is a publicly available resource (https://physionet.org/content/ctu-uhb-ctgdb/1.0.0/). Data from the JNU-CTG database are available from the authors upon reasonable request.
